# Tissue-specific transcriptional programming of macrophages controls the microRNA transcriptome targeting multiple functional pathways

**DOI:** 10.1016/j.jbc.2024.107244

**Published:** 2024-03-29

**Authors:** Magdalena A. Czubala, Robert H. Jenkins, Mark Gurney, Leah Wallace, Benjamin Cossins, James Dennis, Marcela Rosas, Robert Andrews, Donald Fraser, Philip R. Taylor

**Affiliations:** 1 Systems Immunity Research Institute and Division of Infection and Immunity, Cardiff University, Cardiff, UK; 2 Wales Kidney Research Unit, Cardiff University, Cardiff, UK; 3 UK Dementia Research Institute at Cardiff, Cardiff University, Cardiff, UK

**Keywords:** microRNA (miRNA), macrophage, GATA transcription factor, homeostasis, transcriptomics

## Abstract

Recent interest in the biology and function of peritoneal tissue resident macrophages (pMΦ) has led to a better understanding of their cellular origin, programming, and renewal. The programming of pMΦ is dependent on microenvironmental cues and tissue-specific transcription factors, including GATA6. However, the contribution of microRNAs remains poorly defined. We conducted a detailed analysis of the impact of GATA6 deficiency on microRNA expression in mouse pMΦ. Our data suggest that for many of the pMΦ, microRNA composition may be established during tissue specialization and that the effect of GATA6 knockout is largely unable to be rescued in the adult by exogenous GATA6. The data are consistent with GATA6 modulating the expression pattern of specific microRNAs, directly or indirectly, and including miR-146a, miR-223, and miR-203 established by the lineage-determining transcription factor PU.1, to achieve a differentiated pMΦ phenotype. Lastly, we showed a significant dysregulation of miR-708 in pMΦ in the absence of GATA6 during homeostasis and in response to LPS/IFN-γ stimulation. Overexpression of miR-708 in mouse pMΦ *in vivo* altered 167 mRNA species demonstrating functional downregulation of predicted targets, including cell immune responses and cell cycle regulation. In conclusion, we demonstrate dependence of the microRNA transcriptome on tissue-specific programming of tissue macrophages as exemplified by the role of GATA6 in pMΦ specialization.

Tissue resident macrophages (resMΦ) are present in all vertebrate tissues and share core functions as modulators of tissue immune responses and integral components of homeostatic physiology. PU.1 is a lineage-determining transcription factor, which orchestrates gene expression and chromatin accessibility in prototype macrophages to define their core functions (1). Within the tissue, prototype macrophages acquire tissue-specific properties due to collaborative and hierarchical interactions of PU.1 and tissue-specific factors, such as GATA-binding factor 6 (GATA6) in tissue-resident peritoneal macrophages (pMΦ). Indeed, GATA6 is required for the regulation of anatomical localization of pMΦ, generation of tissue macrophage diversity, appropriate immune response to lipopolysaccharide (LPS), and pMΦ proliferative renewal (2, 3, 4, 5).

Mature miRs are 18 to 24 nucleotide ssRNA molecules often conserved between species (6). Pre-miR are transcribed from DNA sequences into primary miR and processed further into their mature form annotated as either -5p or -3p, depending on the alignment of the primary transcript. These miRs use their “seed sequence” and other pairing mechanisms to target specific mRNA leading to translational regulation or transcript degradation (reviewed in (7)). miR-dependent posttranscriptional gene regulation is not fully understood; however, previous studies identified the subcellular localization and abundance of miR as factors contributing to this process (7). miR plays an important role in various aspects of macrophage biology, such as cholesterol efflux, cell polarization, and immune responses (8, 9, 10). *Gata6* itself has been shown to be a target of multiple miR (11). However, miR transcriptome and its dependence on tissue-specific macrophage programming, such as effected by GATA6, has not been explored. Here, we demonstrate a significant disturbance in the miR composition of primary pMΦ in mice with a myeloid deficiency of *Gata6*, predicted to influence multiple biological functions. Using miR-708 as an example, we demonstrate its dysregulation in pMΦ lacking GATA6, identifying it as an anti-inflammatory miR and characterizing its target genes and pathways in *in vivo* pMΦ at homeostasis.

## Results

### GATA6 dictates tissue-specific pMΦ miR transcriptome predicted to affect multiple biological functions

**Figure 1:**
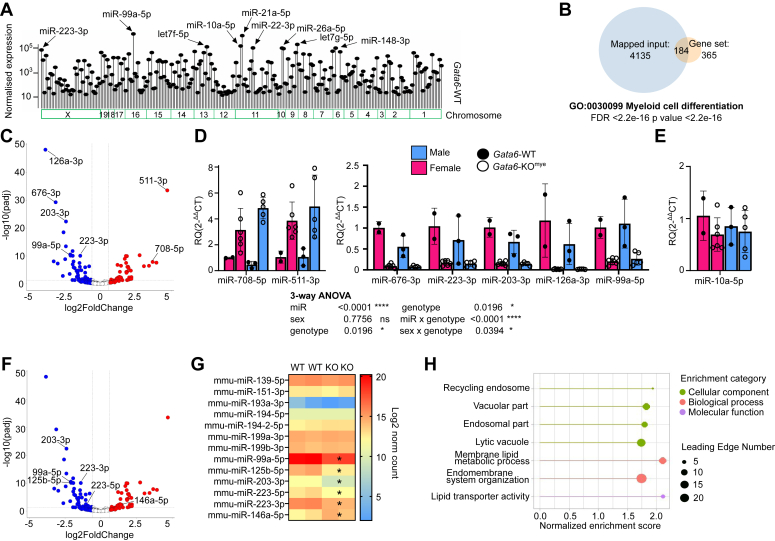
**Tissue-resident peritoneal MΦ microRNA transcriptome is distorted in the absence of GATA6.***A*, small RNA sequencing of *Gata6*-WT and *Gata6*-KO^mye^ pMΦ (*n* = 2, *per genotype*) showing *Gata6*-WT miR transcriptome with highest expressed miRs indicated. Chromosomal location of miRs is indicated in the *bottom* panel (Chromosome). *B*, venn diagram showing one of the significant GO pathways predicted to be modulated by *Gata6*-WT miR transcriptome. *C*, volcano plot showing differential expression of miRs in *Gata6*-WT and *Gata6*-KO^mye^ pMΦ. *D* and *E*, pMΦ sorted to minimum 95% purity from female (*pink*) and male (*blue*) *Gata6*-WT (*closed circle*) and *Gata6*-KO^mye^ (*open circle*) mice (aged 8–12 weeks) were analyzed by RT-qPCR to confirm selected miR expression from small RNA sequencing. miR-10a-5p (*E*) serves as not significantly changed control. Data normalized to average *Gata6*-WT female ΔCT (*n* ≥ 2). Data were analyzed using 3-way Anova. *F* and *G*, volcano plot and a heatmap of pMΦ miRs with indicated signature miRs^4^ expression (log2 normalized count) in *Gata6*-WT and *Gata6*-KO^mye^ pMΦ. *H*, list of pathways from four enrichment categories predicted to be affected by genes targeted by the most abundant and significantly altered miRs from small RNA sequencing data. Results are expressed as the mean ± SD; all RT-qPCR shows independent animal data. miR significantly altered in *Gata6*-KO^mye^ pMΦ marked as “∗”. GATA6, GATA-binding factor 6; pMΦ, tissue-resident peritoneal macrophage.

Investigation into the role of GATA6 transcription factor in pMΦ identified its crucial functions in shaping macrophage metabolism, proliferation, and immune responses (2, 3, 4, 5). Expecting that many of the transcriptional signatures in resident MΦ may be secondary to this transcriptional control and mediated by altered regulation of epigenetic programming, we conducted detailed small RNA-seq of pMΦ from mice lacking functional GATA6 in myeloid cells (*Gata6*-KO^mye^) and their WT counterparts (*n = 2* per genotype) and performed differential expression analysis using DEseq (please refer to experimental procedures for details). We confirmed widespread alterations in miR expression but notably no significant changes in other small RNA molecules (snoRNA and snRNA, etc.) demonstrating the specificity of the changes in miR composition. The mature miR transcriptome of *Gata6*-WT pMΦ consists of 262 entries with high miRBase annotation confidence (selection criteria: ≥10 mean norm expression in *Gata6*-WT, mature miR, miR in repeated locations excluded) (Fig. 1*A* most highly expressed miRs annotated, Table S1). To understand the role of miR transcriptome in pMΦ biology, we performed over representation enrichment analysis of predicted target genes (total 5185) of *Gata6*-WT miR transcriptome matched to the mRNA expressed in *Gata6*-WT microarray (2). As expected, from the high number of predicted genes, the analysis identified multiple biological processes (Table S2). Interestingly, one of the top pathways was “myeloid cell differentiation” (GO:0030099) (Fig. 1*B*), consistent with the involvement of miRs in the regulation of pMΦ specialization.

We identified 40 significantly downregulated and 30 upregulated miRs (adjusted *p* value < 0.05, miRBase high annotation confidence) in *Gata6*-KO^mye^ pMΦ compared to the WT controls with validation of selected miR by RT-qPCR with 100% concordance (Fig. 1, *C* and *D*). Among those, miR-708-5p and miR-511-3p constituted top fold upregulated miRs (28- and 33-fold change, respectively) and the most downregulated miRs included miR-126a-3p (15.7-norm. counts fold change) and miR-676-3p (9.4-fold). There were no statistically significant expression differences between the sexes among investigated miRs (Fig. 1*D*). miR-10a-5p serves as not significantly changed control, females *p* value = 0.250, males *p* value = 0.5714 (Fig. 1*E*). Recently, miR signatures for specific tissue resMΦ have been described (12), and we demonstrate that a specific component of this is partially dysregulated in the absence of GATA6 (Fig. 1, *F* and *G*). Additionally, despite significant downregulation in *Gata6*-KO^mye^ pMΦ, some miRs, such as miR-99a-5p (average norm count, WT = 1.14e-6, KO = 2.49e-5, -4.5 norm. counts FC) (Table S1), remained among the top expressed miRs (Fig. 1, *A* and *C*). To investigate the potential phenotypic impact of those miRs that remain highly abundant in pMΦ despite significant dysregulation in the absence of GATA6, we identified predicted target genes for miR-99a-5p, miR-125b-5p, let-7c-5p, miR-223-5p, miR-221-3p, and miR-146a-5p. This generated 788 protein-coding transcripts predicted by at least two out of three of the algorithms, including TargetScanMouse8.0, miRDB, and DIANA and matched to previously published microarray from these cells whose expression was altered by >20% in the absence of GATA6 (2). We used a minimum 20% expression change because this is consistent with typically quite modest miR-mediated repression (13, 14) (Table S3). GSEA analysis of the targets identified multiple biological processes and molecular functions including membrane lipid metabolic processes and lipid transporter activity of the *Gata6*-KO^mye^ pMΦ (Fig. 1*H*, Table S3). In support of these findings, *Gata*6-KO^mye^ pMΦ were previously described to have altered lipid metabolism (5), and GATA6 depletion led to the accumulation of lipid vacuoles in sebocytes (15). Membrane lipids, such as sphingolipids, play an important role in the induction of inflammation via, among other pathways, activation of COX-2 and synthesis of prostaglandins (16, 17). Therefore, it is likely that investigated miRs might have a pronounced effect on pMΦ immune responses via the regulation of lipid metabolism. Interestingly, we also identified 93 transcription regulators (*e.g.*, Maf, Meis1, and Fos were all significantly changed in *Gata6*-KO^mye^ pMΦ) predicted to be targeted by these highly abundant miRs, implying a potentially broader indirect effect on pMΦ biology (Table S3).

**Figure 2:**
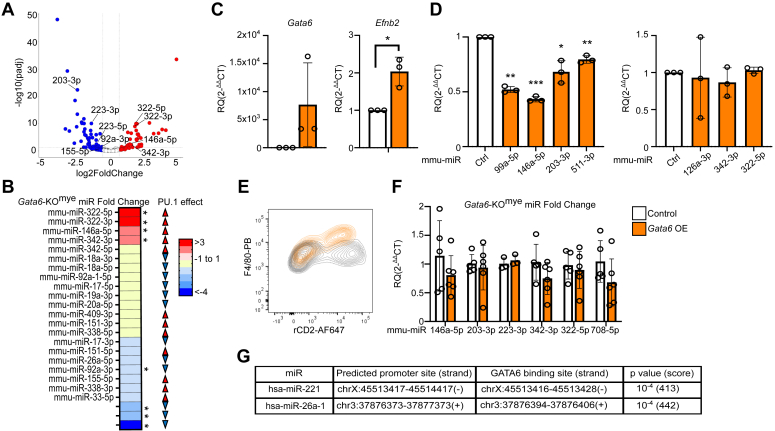
**Overexpression of Gata6 in BMDM and *Gata6*-KO**^**mye**^**peritoneal MΦ does not restore miR transcriptome.***A* and *B*, volcano plot and a heatmap showing fold change expression of known PU.1-modulated miRs in *Gata6*-KO^mye^ pMΦ. Arrows indicate reported effect of PU.1 on miR expression. *C*, expression of *Gata6*, *Efnb2* and (*D*) selected miRs in *Gata6*-KO^mye^ BMDM infected with *Gata6* overexpressing (*orange* bars) or Ctrl (*white* bars) lentivirus for 5 days (*n* = 3). miRs unaffected by *Gata6* overexpression are shown in the *right* graph. *E* and *F*, contour plot showing increase of F4/80 surface expression on *in vivo* transduced *Gata6*-KO^mye^ pMΦ with *Gata6* overexpressing (rCD2+, *orange*) versus Ctrl (*gray*) lentivirus and RT-qPCR analysis of expression levels of selected miRs in these cells (*n* ≥ 3). *G*, a table showing selected miRs promoter sites and potential GATA6-binding site with strands (− or +) indicated in the brackets. Results are expressed as the mean ± SD; all RT-qPCR shows results from independent mice. Data were analyzed with paired (*C*, *D*) or unpaired (*F*) *t* test. *p* < 0.05 was considered statistically significant (∗*p* < 0.05, ∗∗*p* < 0.01, ∗∗∗*p* < 0.001). BMDM, bone marrow–derived macrophage; GATA6, GATA-binding factor 6; pMΦ, tissue-resident peritoneal macrophage.

Considering the potential role of miRs on myeloid cell differentiation (Fig. 1*B*), we compared *Gata6*-KO^mye^ pMΦ miR transcriptome to those known to be regulated by PU.1 (18) (Fig. 2, *A* and *B*, Table S1). We observed an overlap between some of PU.1 miR targets and miRs altered in GATA6-deficient pMΦ, including miR-203-3p, 223-5p, and 146a-5p, which were also recently identified as part of the pMΦ miR-signature (12). Thus, GATA6 may contribute to the PU.1-regulated miR signature in tissue-specialized pMΦ. To investigate this concept, we induced *Gata6* expression in *Gata6*-KO^mye^ bone marrow–derived macrophages (BMDM) (Fig. 2, *C* and *D*) and *in vivo* in pMΦ (Fig. 2, *E* and *F*) as previously described (2, 19). *Gata6*-expressing BMDM showed significantly increased expression of positive control gene *Efnb2* and reduced levels of miR-146a-5p and 511-3p in concordance with the expectation from miR-seq (Fig. 2, *C* and *D*). Interestingly, miR-99a-5p and 203-3p were also significantly downregulated, while other investigated miRs remained unchanged (Fig. 2*D*), in contrast to data obtained from *Gata6*-KO^mye^ pMΦ miR-seq data. This demonstrates a complex regulation of miRs with differential outcomes in different macrophage cell models and/or differentiation states. To determine whether GATA6 alone controlled microRNA profile in pMΦ, we employed lentiviral vectors to transduce *Gata6* for 4 days into established *Gata6*-KO^mye^ pMΦ *in vivo* (19). We have previously shown that GATA6 regulates the expression of pMΦ surface marker F4/80 (2). As expected, lentivirally transduced population demonstrated a predicted increase of F4/80 expression (Fig. 2*E*), signifying the restoration of GATA6 activity. However, it failed to significantly alter miR expression in a manner congruent with the miR-seq data (Fig. 2*F*). Thus, GATA6 programming of pMΦ regulates the miR transcriptome, most likely at early stages of macrophage tissue specialization, and *via* both direct and indirect secondary effects. To anticipate which miRs might be directly responsive to an acute expression of GATA6, we employed predicted putative promoters for hsa-miRs identified and validated previously by two different algorithms (20, 21). We focused on miRs which had the highest FC between *Gata6*-WT and -KO^mye^ pMΦ and were most robustly expressed in *Gata6*-WT: miR-99a, miR-676, miR-221, miR-130b, miR-218-1, miR-200b, miR-200a, miR-100, miR-27a, let-7f-1, miR-10a, miR-148a, miR-26a-1, and miR-192. We then used UCSC Genome Browser and integrated Jasper transcription factor–binding site prediction tool (with TFBS predictions selected with a PWM relative score >0.8 and *p* value < 10^−4^, corresponding to a score above 400) to identify potential binding sites for GATA6. From the investigated miR, only predicted promoters for hsa-miR-26a-1 and hsa-miR-221 showed the presence of a potential GATA6-binding site within or in the proximity of the predicted promoter (Fig. 2*G*). This suggests that many miRs dysregulated in *Gata6*-KO^mye^ pMΦ might be an indirect consequence of the lack of GATA6 functionality.

### miR-708 is downregulated by LPS/IFN-γ and regulates innate immune response pathways

**Figure 3:**
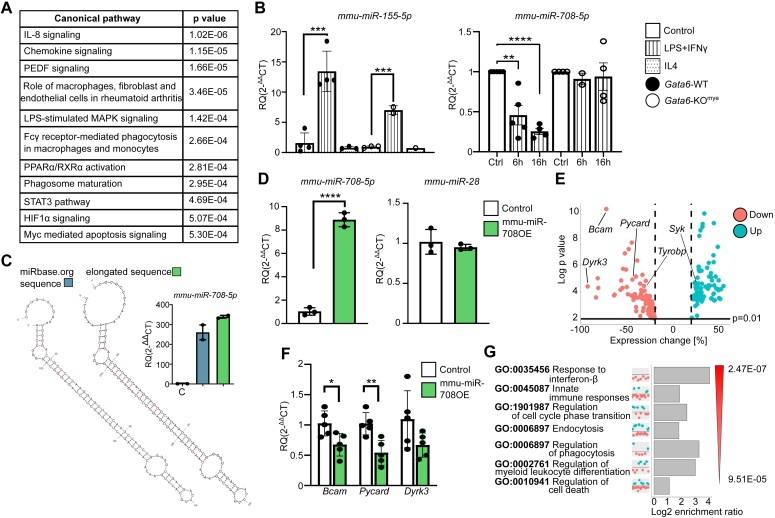
**miR708 is potentially involved in regulation of immune responses in peritoneal MΦ.***A*, some of the top significant canonical pathways predicted to be affected by GATA6-regulated miRs, as indicated by IPA software analysis. *B*, pMΦ freshly isolated from *Gata6*-WT or -KO^mye^ mice stimulated *in vitro* with LPS (100 ng/ml) and IFN-γ (20 ng/ml) or IL4 (20 ng/ml) for indicated times. Expression level of *mmu-miR-155-5p* and *mmu-miR-708-5p* was measured by RT-qPCR. Analysis was normalized to control-treated sample (*n* ≥ 2) and to U6 endogenous expression. *C*, secondary structure prediction (MFold) of primary mmu-miR-708 sequence from miRbase.org*versus* sequence with additional six nucleotides used in this study and miR-708-5p overexpression efficiency of the above sequences in BMDM. “C” - control. *D*, expression of miR-708-5p and miR-28 in sorted C57BL/6 pMΦ overexpressing mir-708 (*green circles*) or control (*black circles*) for four days *in vivo*^6^. *E*, the volcano plot showing genes significantly changed in miR-708 overexpressing C57BL/6 pMΦ. *F*, RT-qPCR (SYBR *Green*) confirming expression change of selected genes in C57BL/6 pMΦ overexpressing mir-708 (*green* bars) or Ctrl (*black* bars). *G*, IPA analysis of GO terms for significantly altered genes in miR-708 overexpressing pMΦ. Results are expressed as the mean ± SD; all RT-qPCR shows independent replicate data. Data were analyzed with unpaired *t* test (*B*, *D*, and *F*). BMDM, bone marrow–derived macrophage; GATA6, GATA-binding factor 6; IFN-γ, interferon gamma; LPS, lipopolysaccharide; pMΦ, tissue-resident peritoneal macrophage.

To understand which pathways are affected due to dysregulated miR transcriptome caused by GATA6 deficiency, we matched predicted gene targets of significantly changed microRNAs with significantly altered mRNAs from *Gata6*-WT and *Gata6*-KO^mye^ bulk sequencing (2) using IPA software. This led to a list of 311 candidate genes (Table S2) involved in 408 canonical pathways, including pathways related to macrophage biology and immune activation (Fig. 3*A*). Therefore, we investigated miR regulation in pMΦ under inflammatory conditions. *Gata6*-WT and *Gata6*-KO^mye^ pMΦ were treated *in vitro* with LPS/interferon gamma (IFN-γ) or IL4 for 6 and 16 h. We confirmed the expected upregulation of miR-155 in LPS/IFN-γ–challenged pMΦ (22) in both genotypes (Fig. 3*B*). Interestingly, we identified a significant decrease in miR-708-5p in *Gata6*-WT, but not in -KO^mye^ pMΦ, as soon as 6 h post treatment that remained low after 16 h (Fig. 3*B*). An anti-inflammatory role of miR-708 has been previously demonstrated (23), consistent with its downregulation in *Gata6*-WT pMΦ in response to inflammatory stimuli. miR-708 is one of the most highly upregulated miRs in *Gata6*-KO^mye^ pMΦ (28-fold increase) (Table S1) and did not downregulate in *Gata6*-KO^mye^ cells after LPS/IFN-γ exposure. This distorted miR response in *Gata6*-KO^mye^ pMΦ could partially contribute to the previously determined disrupted immune activation evident in these cells (5).

To explore this, we employed lentiviral vectors to overexpress murine miR-708 *in vivo* in C57BL/6 pMΦ. For this purpose, the pre-miR-708 sequence deposited in miRbase was elongated by six nucleotides from the genomic sequence to create a short single stranded 3′ tail (Fig. 3*C*). The single stranded tails are required for correct cleavage by the RNase III-type endonucleases Drosha and Dicer to produce the mature-miR (13). Indeed, the elongated sequence demonstrated robust high expression efficiency in BMDM (Fig. 3*C*). First, we showed successful overexpression of the dominant mature miR-708-5p (8.54-fold expression change, *p*-value < 0.0001) in *in vivo* pMΦ and confirmed that it had no effect on the expression of miR-28, which shares the same seed sequence (Fig. 3*D*). Transcriptome analysis of C57BL/6 pMΦ overexpressing miR-708 revealed 167 significantly changed protein-coding transcripts (75 up and 92 down) (*p*-value <0.01 and min 20% expression change consistent with typically quite modest miR-mediated repression, which is often less than 20% (13, 14)) (Fig. 3*E* and Table S4). These included 33- and 13- predicted targets for both mmu-miR-708-3p and mmu-miR-5p, respectively, as specified by TargetScanMouse8.0, miRDB, and DIANA algorithms. We further validated the regulation of some genes in pMΦ samples from independent *in vivo* miR-708 overexpression experiments (Fig. 3*F*). miR activity was suggested to be dependent on its abundance (12). Overexpression of miR-708 in our setting mirrored the fold-change difference of this miR observed between *Gata6*-WT and -KO^mye^ pMΦ, suggesting that observed mRNA targets are regulated by changes in miR-708 abundance relevant to physiological conditions. Using a recently published target prediction resource (12) with an incorporated abundance threshold for miRs, we confirmed effective targeting of *Bcam*, *Pycard,* and *Dyrk3* in pMΦ by miR-708-5p expressed at physiological levels, therefore further validating our results. GO analysis of miR-708 targets indicated involvement of this miR in pathways regulating immune responses, cell cycle, and cell death (Fig. 3*G*). Therefore, we provide novel data on gene regulation in pMΦ downstream of miR-708, which supports the role of miR-708 in the regulation of macrophage inflammatory phenotype.

## Discussion

Complete understanding of the mechanisms that control pMΦ functions and development in tissue-specific microenvironments remains a focal question in the macrophage biology field, despite remarkable advances made in recent years (2, 3, 4, 24, 25). With growing insight into the very complex mechanisms and functions of miR in cells (7, 12, 14), comes an appreciation of their importance in directing functional outcomes of gene expression. Here, we demonstrate that the programming of pMΦ by GATA6 dictates miR profile and that lack of GATA6 leads to a disturbed transcriptome with potential functional consequences. Small RNA-seq of pMΦ from *Gata6-*WT mice revealed complex homeostatic miR transcriptome of these cells, consisting of 262 miR expressed at various abundance. This included previously described core peritoneal macrophage miR, miR-199a/b-3p, miR-203-3p, and miR-99a-5p, the latter expressed at particularly high levels (12). Over representation enrichment analysis performed on predicted target genes for *Gata6*-WT miR transcriptome identified numerous pathways involved in cell differentiation, lipid metabolism, and immune responses. Although current prediction algorithms are becoming increasingly accurate (26), they can over or underestimate the targeting potential of miR. However, the analysis highlights the potentially vast scope of the functions regulated by miR transcriptome in these cells, with the importance of some individual miR previously documented (27, 28). While our computational analysis suggests potential miRs of interest, it remains to be determined which miRs are under the direct control of GATA6 or other transcription factors dysregulated in *Gata6*-KO^mye^ pMΦ (2).

PU.1 is involved in the differentiation and maturation of pMΦ, partially by the modulation of miR transcriptome of the progenitor cell (18). Hierarchical action of peritoneal tissue-specific GATA6 further specializes macrophages in the peritoneal cavity. Here, we demonstrated that some of the GATA6-induced tissue specialization is potentially driven by modulation of the miR profile established by PU.1. In total, six miR targeted by PU.1 were significantly changed in pMΦ from *Gata6*-KO^mye^ mice, suggesting that GATA6 might act, most likely indirectly, to downregulate miR-322, miR-146a, and miR-342 and upregulate miR-92a, miR-223, and miR-203 patterns established during differentiation to achieve terminal pMΦ phenotype. The effect of these changes on miR target gene translation requires further evaluation to fully understand the mechanism and importance behind these alterations.

Deletion of functional GATA6 in pMΦ, using the *Gata6*-KO^mye^ mouse model (2, 3, 4), revealed statistically significant disruption in 70 miR with the predicted role in immune responses of cells. We (2, 5) and others (3, 4) have previously described dysregulated immune functions of *Gata6*-KO^mye^ pMΦ. Our new data suggest that these alterations could be in part dictated by altered miR-dependent translational control of gene expression in these cells. Interestingly, although GATA6 appeared necessary for the pMΦ miR signature that we have uncovered, we failed to fully restore it in *Gata6*-KO^mye^ pMΦ upon GATA6 overexpression. This suggests that miR profile may be established at the earlier stages of tissue macrophage specialization or that a more complex regulatory network is responsible for the induction of these miRs. Rose, *et al* 2021, has previously described multileveled control of the expression of specific immune-functioning miR, including cis-regulatory elements and chromatin accessibility of the finally differentiated cells (12). Indeed, activity of some miR promoters can be restricted to specific developmental stages of the cells (12). miR-99a-5p has been previously implicated in the regulation of tumor necrosis factor alpha in macrophages *in vivo* and LPS/IFNγ-induced bactericidal activity in bone BMDM (29). Among other miRs, miR-99a-5p remained highly abundant in pMΦ despite significant modulation in the absence of GATA6. Our analysis suggested involvement of these miRs’ predicted targets in lipid metabolism and cell membrane rearrangement processes, which are important for mediating a wide range of cellular immune responses (reviewed in (30)). We have previously shown that *Gata6*-KO^mye^ pMΦ display polyploidy (2) and altered regulation of interleukin 1 beta release, latter caused by dysregulated prostacyclin production (5). Thus, even with abundant expression, changes in investigated miRs in *Gata6*-KO^mye^ pMΦ might be sufficient to impose notable phenotypic changes. The actual scale of these changes in relation to miR levels should be further explored.

Our miR analysis combined with mRNA expression data from *Gata6*-KO^mye^ pMΦ (2) confirmed alteration in multiple pathways involved in immune responses of macrophages. Through the study of the dysregulated miR, we uncovered novel inflammatory regulation of miR-708-5p in pMΦ that differed significantly between genotypes. Studies of miR-708 in both macrophage-like and non-immune cell lines identified the roles of this miR in the regulation of tumor necrosis factor alpha/interleukin 1 beta, arachidonic acid pathways and inflammatory responses to *mycobacterium tuberculosis* (23, 31). Interestingly, miR-708-5p decreased in response to LPS/IFNγ in *Gata6*-WT pMΦ but it remained at the homeostatic level in *Gata6*-KO^mye^ pMΦ. This potentially contributes to the disrupted immune responses in these cells (5). While proposed that only the most abundant miR within a cell mediate significant target suppression (32), our data suggests that miR-708 overexpressed to the physiologically relevant levels observed in *Gata6*-KO^mye^ pMΦ, demonstrated functional impact, despite very low abundance compared to other miR present in these cells. We identified a total of 167 significantly changed mRNA in pMΦ overexpressing miR-708 accordingly to selection criteria described. We validated a significant decrease of two of these genes, Bcam and Pycard using RT-QPCR. Although a significant decrease in the level of Dyrk3 mRNA was not confirmed. Over representation enrichment analysis provided support to the immune-regulatory role of miR-708 in pMΦ, to our knowledge, a first report demonstrating this relationship in primary macrophages.

In summary, our analysis of miR profiles using Gata6-KO^mye^ pMΦ as a model of a tissue-resident MΦ that failed to specialize in its tissue microenvironment showed a marked specific dysregulation of miR that was not evident with other small RNA species. This demonstrated an important specialization of the tissue MΦ miR transcriptome during tissue-specific programming. Enrichment analysis of putative target mRNA indicated that the dysregulated miR had the potential to regulate broad pathways of cellular function, which we validated by establishing the impact of miR-708 dysregulation on the transcriptome of pMΦ. We demonstrated, as predicted, that miR-708 targets immune response genes and also transcripts involved in cell cycle and cell death. Together, our data demonstrate that tissue specialization of pMΦ is associated with the acquisition of a specific miR transcriptome that contributes to the biology and immune responses of these cells.

## Experimental procedures

### Ethics

All experiments were approved by the Animal Welfare and Ethical Review Body (AWERB), under the oversight of the Biological Standards Committee. Experiments strictly adhered to the guidelines set forth by the UK Home Office and the Animal [Scientific Procedures] Act 1986, in accordance with EU Directive 2010/63/EU on the protection of animals used for scientific purposes.

### microRNA transcriptome selection criteria

The following criteria were applied to qualify miR for the transcriptome: 1. expression ≥10 normalized counts (data normalized in DESeq2 using scaling factors) in both *Gata6*-WT samples. 2. Mean normalized expression for *Gata6*-WT samples was ≥ 10. 3. Only mature miR were included (3p or 5p), stem loops excluded. 4. miR with MirBase.org high annotation confidence included. 5. miR duplications from multiple genomic locations excluded from Figure 1*A* but retained in Table S1.

### Software

Ingenuity Pathway Analysis package (33), TargetScan (26), Diana microT-CDS (34, 35), miRBase (36), Web-based Gene SeT AnaLysis Toolkit (37), and ShinyGO 0.77 (38) are used.

### Statistical analysis

Data for the experiments were obtained from at least two independent experiments. Data were analyzed as specified for each experiment. For all datasets, *p* < 0.05 was considered as statistically significant (*p* values: ∗<0.05, ∗∗<0.01, and ∗∗∗<0.001). All statistics were performed using GraphPad Prism 9 software (https://www.graphpad.com/). Data presented as the mean ± SD, with super-imposed scatter plot showing independent biological replicates.

## Data availability

Microarray expression data from WT and *Gata6*-deficient tissue-resident peritoneal macrophages (GEO: GSE47049). MicroRNA-sequencing data from WT and *Gata6*-deficient tissue-resident peritoneal macrophages has been assigned ArrayExpress accession E-MTAB-13039. mRNA-sequencing data from C57BL/6 tissue-resident peritoneal macrophages overexpressing mmu-miR-708-5p and control group has been assigned ArrayExpress accession E-MTAB-13782. All other data are contained within this article as supporting information.

## Supporting information

This article contains supporting information (39, 40, 41, 42, 43, 44, 45, 46, 47, 48).

## Conflicts of interest

The authors declare that they have no conflicts of interest with the contents of this article.

## Supporting information

Supplementary Materials

Supplementary Tables Caption
