# Supplementary material for: Tissue-specific transcriptional programming of macrophages controls the microRNA transcriptome targeting multiple functional pathways
Source: J Biol Chem. 2024 Mar 29;300(5):107244. doi: 10.1016/j.jbc.2024.107244 (PMC11067537; doi:10.1016/j.jbc.2024.107244)
Supplement: Supplementary Materials [file mmc5.doc]

**Supplementary experimental procedures**

*Cell culture of bone marrow derived macrophages (BMDM)*

BMDM were derived from bone marrow of *Gata6*-KO^mye^ or C57BL/6 mice in DMEM (supplemented with 4.5 g/L D-glucose, 4 mM L-Glutamine, 10 % (v/v) heat-inactivated fetal bovine serum (FBS), 100 units/mL (v/v) penicillin, 100 μg/mL streptomycin and 20 ng/mL recombinant murine M-CSF (Peprotech) for 7 days as described previously(1).

*Mice and in vivo peritoneal macrophage infection*

All animals were sex-matched and between 8-12 weeks of age at the time of use. Lysozyme M Cre-recombinase “knock-in” congenic mice on CD1 and C57BL/6 background (“*Lyz2^Cre^*, B6.129P2-*Lyz2^tml(cre)lfo/J^*)(2) and conditional “floxed” *Gata6*-deficient mice (“*Gata6^Fl^*, *Gata6^tm2.1Sad/J^*)(3) were obtained from Jackson Labs and bred in our animal facilities as previously described(4). C57BL/6 mice were obtained from Charles Rivers Laboratories. All experiments were not blinded. Intraperitoneal injection of lentiviruses and peritoneal cells collection were described before(5). For immune stimulation experiments, pMΦ were obtained from the peritoneal lavage of *Gata6*-WT and *Gata6*-KO^mye^ mice and plated for 3 h in 24 well plate in DMEM(6). Non-adherent cells were washed out and remaining pMΦ were stimulated with 100 ng/mL ultra-pure LPS-EB from *E.coli* O111:B4 (invivoGen) and 20 ng/mL recombinant murine IFN-γ (Peprotech), 20 ng/mL recombinant murine IL4 (Peprotech) or control (DMEM) for 6 or 16 h. Flow cytometry was performed as described previously(5). Antibodies: F4/80-Pacific Blue (BM8, # 123123 Biolegend), mouse anti ratCD2-Alexa Fluor 647 (BioRad, #MCA154A647). For RNA extraction studies, collected lavages were stained with LIVE/DEAD fixable stain (ThermoFisher), Tim4 and F4/80 and GFP+ or rCD2+ pMΦ were sorted to high purity (>98%) on BD FACSAria III (Becton, Dickinson and Company) prior to RNA extraction.

*Plasmids and reagents*

pHR’SIN-cPPT-SEW plasmid(4,5,7) (encoding enhanced GFP marker) was modified using “in-fusion” cloning kit (Clontech) to express *mmu-miR-708* or *Gata6* as published previously(4). Lentiviral particles were produced as described previously (5) by transfection of HEK293T cells with lentiviral plasmid and helper plasmids, pCMV-ΔR8.91 (Gag/Pol, Tat and REV)(8) and pMD2.G (VSV-G envelop(9)).

*Small RNA and mRNA sequencing*

Total RNA extraction, including small RNA and mRNA was performed using miRNeasy Micro Kit (Qiagen) accordingly to manufacturer’s instructions. Small RNA sequencing was run on Illumina next-generation sequencing (Exigon, Vedbaek Denmark). Transcriptome sequencing of C57BL/6 pMΦ overexpressing mmu-miR-708 or control was performed on Illumina HiSeq3000 using NextSeq 500/550 High Output Kit v2.5 (150 cycles) and NextSeq 500/550 Mid Output Kit v2.5 (150 cycles) (Illumina). Total of 45M reads was obtained from the sequencing. Paired-end reads from Illumina sequencing were trimmed with Trim Galore (10) and assessed for quality using FastQC(11), using default parameters. Reads were mapped to the mouse GRCm38 reference genome using STAR(12) and counts were assigned to transcripts using featureCounts(13) with the GRCm38.84 Ensembl gene build GTF. Both the reference genome and GTF were downloaded from the Ensembl FTP site (14). Differential gene expression analyses used the DESeq2 package(15). Genes were discarded from the analysis differential expression failed to be significant (significance: adj.pval < 0.05, Benjamini-Hochberg correction for multiple testing).

*Preparation of lentiviral vectors and infectious lentiviral particles*

pHR’SIN-cPPT-SEW plasmid(4,5,16) (encoding enhanced GFP marker) was modified using “in-fusion” cloning kit (Clontech) to express mmu-miR-708 or *Gata6* overexpression(4). Lentiviral particles were produced as described previously (5). mmu-mir-708 overexpression sequence (added 6-nucleotide sequence underlined): 5’CTGTGTTTGAAATGGGGACTGCCCTCAAGGAGCTTACAATCTAGCTGGGGGTAGATGACTTGCACTTGAACACAACTAGACTGTGAGCTTCTAGAGGGCAGGGGCCTTAAAAGTT3’ control overexpression vector sequence: 5’GGTAGTGAGTTCGCGATCGTGCATCGATGCACGATCGCGAACTCACTACCTTTTT3’.

*Quantitative real-time PCR (RT-qPCR)*

Total RNA including microRNA was isolated from cells using miRNeasy Micro Kit (Qiagen) accordingly to manufacturer instructions. microRNA expression was measured with Taqman®MicroRNA Assays (Applied Biosystems, Life Technologies) using 10ng of total RNA per microRNA accordingly to manufacturer’s instructions. For mRNA measurements, cDNA was synthesized using Life Technologies high-capacity cDNA reverse transcription kit (ThermoFisher Scientific). mRNA was measured using Power SYBR Green PCR Master Mix (Thermo Fisher Scientific). Both assays were run on ViiA^TM^ 7 Real-time PCR system (Applied Biosystems, Life Technologies). microRNA data was normalised to U6 endogenous control and mRNA to Ywhaz endogenous expression, and to an appropriate control for each experiment. Data expressed as relative quantification (RQ-2^ΔΔ^CT). TagMan microRNA assays (#4427975, LifeTech): miR-10a-5p (assay ID:000387), miR-28-5p (ID:000411), miR-99a-5p (ID:000435), miR-126a-3p (ID:002228), miR-146a-5p (ID:000468), miR-155-5p (assay ID:002571), miR 203-3p (ID:000507), miR-223-3p (assay ID:002295), miR-322-5p(mmu-miR-424) (ID:001076), miR-342-3p (ID:002260), miR-511-3p (ID:463069_mat), miR-676-3p (assay ID: 001959), miR-708-5p (ID:002341), U6 snRNA (ID:001973). Primers sequences: *Ywhaz* 5’-TTGAGCAGAAGACGGAAGGT-3′ and 5′-GAAGCATTGGGGATCAAGAA-3′, *Gata6 forw* 5’-AAAGCTTGCTCCGGTAACAG-3′ and rev 5’-TCTCCCACTGCAGACATCAC-3′, *Dyrk3 forw* 5’-TCGGACACATTCCAGCAACC-3′ and rev 5′-TGAGTGGCTCAGTGGTAATATTTAG-3′, *Bcam* 5’-AGTTTGCCCAGGAGATTGC-3′ and 5′-CCCGTTTCGGTACCATGTGA-3′, *Pycard* forw 5’-GTACAGCCAGAACAGGACA-3′ and rev 5′-CAGCACACTGCCATGCAAA-3′, *Efnb2* 5’-CCAGGAATCACGGTCCA-3′ and 5′-CTGTTGCCATCGGTGCT-3′.

References

1. Davies, L. C. (2014) *Control of Macrophage Homeostasis.*PhD, Cardiff University

2. Clausen, B. E., Burkhardt, C., Reith, W., Renkawitz, R., and Forster, I. (1999) Conditional gene targeting in macrophages and granulocytes using LysMcre mice. *Transgenic Res* **8**, 265-277

3. Sodhi, C. P., Li, J., and Duncan, S. A. (2006) Generation of mice harbouring a conditional loss-of-function allele of Gata6. *BMC Dev Biol* **6**, 19

4. Rosas, M., Davies, L. C., Giles, P. J., Liao, C. T., Kharfan, B., Stone, T. C., O'Donnell, V. B., Fraser, D. J., Jones, S. A., and Taylor, P. R. (2014) The transcription factor Gata6 links tissue macrophage phenotype and proliferative renewal. *Science* **344**, 645-648

5. Ipseiz, N., Czubala, M. A., Bart, V. M. T., Davies, L. C., Jenkins, R. H., Brennan, P., and Taylor, P. R. (2020) Effective In Vivo Gene Modification in Mouse Tissue-Resident Peritoneal Macrophages by Intraperitoneal Delivery of Lentiviral Vectors. *Mol Ther Methods Clin Dev* **16**, 21-31

6. Ipseiz, N., Pickering, R. J., Rosas, M., Tyrrell, V. J., Davies, L. C., Orr, S. J., Czubala, M. A., Fathalla, D., Robertson, A. A., Bryant, C. E., O'Donnell, V., and Taylor, P. R. (2020) Tissue-resident macrophages actively suppress IL-1beta release via a reactive prostanoid/IL-10 pathway. *EMBO J*, e103454

7. Taylor, P. R., Heydeck, D., Jones, G. W., Kronke, G., Funk, C. D., Knapper, S., Adams, D., Kuhn, H., and O'Donnell, V. B. (2012) Development of myeloproliferative disease in 12/15-lipoxygenase deficiency. *Blood* **119**, 6173-6174; author reply 6174-6175

8. Zufferey, R., Nagy, D., Mandel, R. J., Naldini, L., and Trono, D. (1997) Multiply attenuated lentiviral vector achieves efficient gene delivery in vivo. *Nat Biotechnol* **15**, 871-875

9. Naldini, L., Blomer, U., Gallay, P., Ory, D., Mulligan, R., Gage, F. H., Verma, I. M., and Trono, D. (1996) In vivo gene delivery and stable transduction of nondividing cells by a lentiviral vector. *Science* **272**, 263-267

10. Babraham Institute. (2023) Babraham Bioinformatics - Trim Galore.

11. Babraham Institute. (2023) Babraham Bioinformatics - FastQC.

12. Dobin, A., Davis, C. A., Schlesinger, F., Drenkow, J., Zaleski, C., Jha, S., Batut, P., Chaisson, M., and Gingeras, T. R. (2013) STAR: ultrafast universal RNA-seq aligner. *Bioinformatics* **29**, 15-21

13. Liao, Y., Smyth, G. K., and Shi, W. (2014) featureCounts: an efficient general purpose program for assigning sequence reads to genomic features. *Bioinformatics* **30**, 923-930

14. Ensembl. (2023) FTP Download.

15. Love, M. I., Huber, W., and Anders, S. (2014) Moderated estimation of fold change and dispersion for RNA-seq data with DESeq2. *Genome Biol* **15**, 550

16. Demaison, C., Parsley, K., Brouns, G., Scherr, M., Battmer, K., Kinnon, C., Grez, M., and Thrasher, A. J. (2002) High-level transduction and gene expression in hematopoietic repopulating cells using a human immunodeficiency [correction of imunodeficiency] virus type 1-based lentiviral vector containing an internal spleen focus forming virus promoter. *Hum Gene Ther* **13**, 803-813
