# Supplementary material for: Tissue-specific transcriptional programming of macrophages controls the microRNA transcriptome targeting multiple functional pathways
Source: J Biol Chem. 2024 Mar 29;300(5):107244. doi: 10.1016/j.jbc.2024.107244 (PMC11067537; doi:10.1016/j.jbc.2024.107244)
Supplement: Supplementary Tables Caption [file mmc6.docx]

**Supplementary Table 1**

MiR sequencing differential expression analysis (DEseq) of *Gata6*-WT and *Gata6*-KO^mye^ pMΦ. miR allocated to WT transcriptome are indicated in column T (Status). miRbase annotation confidence and dominant strain of each miR are shown. MiR appearing in multiple chromosomal locations are included.

**Supplementary Table 2**

List of predicted target genes of *Gata6*-WT pMΦ miR transcriptome matched to the mRNA expressed in *Gata6*-WT pMΦ microarray (2). High confidence and experimentally observed interactions included only. Genes targeted by multiple miR are repeated in consequent rows with corresponding information. Sheet 2 contains the over representation enrichment analysis (ORA) of the predicted target genes. Myeloid cell differentiation pathway is highlighted in green and the genes matched to the gene set are listed.

**Supplementary Table 3**

Predicted target genes for selected, significantly downregulated and remaining the top expressed miR in *Gata6*-KO^mye^ pMΦ (miR-99a-5p, -125b-5p, let-7c-5p, -223-5p, -221-3p and -146a-5p). Target genes were predicted by at least 2 out of 3 algorithms, including TargetScanMouse8.0, miRDB and DIANA and matched to previously published microarray from these cells whose expression was altered by >20% in the absence of GATA6 (2). Gene Set Enrichment Analysis (GSEA) analysis of these genes in the following enrichment categories: geneontology biological process noRedundant, geneontology molecular function noRedundant, geneontology cellular component noRedundant and KEGG is shown. Pathways visualized in Figure 1H are highlighted in green.

**Supplementary Table 4**

mRNA sequencing results from C57BL/6 pMΦ transfected with lentivirus overexpressing mmu-miR-708 or control construct for 4 days *in vivo*. Gene list selected by p-value <0.01 and min 20% expression change compared to control samples. Predicted target genes for mmu-miR-708 -5p and -3p by each of the algorithm (TargetScan (T), Diana (D) and mirDB(M)) are indicated.
